# Roles of the Adenosine Receptor and CD73 in the Regulatory Effect of γδ T Cells

**DOI:** 10.1371/journal.pone.0108932

**Published:** 2014-09-30

**Authors:** Dongchun Liang, Aijun Zuo, Hui Shao, Mingjiazi Chen, Henry J. Kaplan, Deming Sun

**Affiliations:** 1 Doheny Eye Institute, Department of Ophthalmology, University of California Los Angeles, Los Angeles, California, United States of America; 2 Department of Ophthalmology and Visual Sciences, Kentucky Lions Eye Center, University of Louisville, Louisville, Kentucky, United States of America; Oregon Health & Science University, United States of America

## Abstract

The adenosine A2A receptor (A2AR), the main functional adenosine receptor on murine T cells, plays a unique role in the attenuation of inflammation and tissue damage in vivo. Here, we showed that, of the immune cell types tested, activated γδ T cells expressed the highest levels of A2AR mRNA and that A2AR ligation inhibited αβ T cell activation, but enhanced γδ T cell activation. We also showed that the inhibitory effect of an adenosine receptor agonist on autoreactive T cells was prevented by addition of a low percentage of activated γδ T cells. Furthermore, compared to resting cells, activated γδ T cells expressed significantly lower levels of CD73, an enzyme involved in the generation of extracellular adenosine. Exogenous AMP had a significant inhibitory effect on autoreactive T cell responses, but only in the presence of CD73^+^ γδ T cells, and this effect was abolished by a CD73 inhibitor. Our results show that expression of increased amounts of A2AR allows γδ T cells to bind adenosine and thereby attenuate its suppressive effect, while decreased expression of CD73 results in less generation of adenosine in the inflammatory site. Together, these events allow activated γδ T cells to acquire increased proinflammatory activity, leading to augmented autoimmune responses.

## Introduction

Adenosine accumulates at inflamed sites as a result of release of adenosine triphosphate (ATP) into the extracellular environment, its subsequent dephosphorylation to adenosine diphosphate (ADP) and adenosine monophosphate (AMP), and a terminal reaction in which AMP is converted to adenosine [1], [2]. Under stress conditions, adenosine release in damaged tissues decreases the energy demand of the tissue by exerting a direct inhibitory effect on parenchymal cell function [1], [3], [4]. In addition, it also reduces the local inflammatory response and modulates various immune responses [5]–[7]. Release of adenosine and its binding to adenosine receptors (ARs) on immune cells represents a potent endogenous immunosuppressive pathway that regulates the immune response to harmful external insults [8]. Multiple lines of evidence have shown that extracellular adenosine, acting via the adenosine A2A receptor (A2AR), is an important negative regulator of T cell development and function [3], [6], [9]–[11]. However, a proinflammatory effect of adenosine has also been recognized [12]–[14].

A regulatory effect of γδ T cells on adaptive immunity has been repeatedly observed [15]–[18], but how these cells regulate the immune response is poorly understood, and how they enhance an immune response in some cases, but inhibit it in others, remains largely obscure. Our previous studies have shown that the regulatory effect of γδ T cells depends on their activation status and that a large proportion of γδ T cells from immunized B6 mice are activated, whereas most γδ T cells from naïve mice are not (resting cells) [19], [20]. Moreover, many factors, such as cytokines and Toll-like receptor (TLR) ligands, can increase γδ T cell activation in the absence of TCR ligation, leading to an enhanced proinflammatory effect of γδ T cells [19]–[22]. To better understand the mechanisms by which γδ T cells regulate Th17 responses, we looked for molecules that cause γδ T cell activation in vivo. In this study, we showed that γδ T cell-mediated immunoregulation was strongly affected by the interaction of these cells with adenosine or AR agonists. Adenosine can bind to four different types of ARs, designated A1R, A2AR, A2BR, and A3R [3], [5], [23], [24], and it has long been recognized that adenosine suppresses T cell activity primarily by acting on A2ARs [9], [25]–[29]. In our study, we found that, although AR agonists had a strong suppressive effect on αβ T cell activation, their effect on γδ T cells was stimulatory, rather than inhibitory. AR agonists enhanced the Th17 response by activating γδ T cells, which converted the anti-inflammatory effect of adenosine on the Th17 response into a proinflammatory effect. Of the immune cell types tested from mice immunized with a uveitogenic antigen to induce uveitis, activated γδ T cells expressed the highest levels of A2AR, allowing them to competitively bind adenosine generated in inflamed tissues, leading to increased activation of γδ T cells and Th17 autoreactive T cells.

We also examined the role of the key adenosine generating enzyme, CD73, a glycosyl phosphatidylinositol-linked membrane protein that catalyzes the extracellular dephosphorylation of AMP to adenosine [30], [31]. Our studies showed that CD73 expressed on γδ T cells was more functionally active than that expressed on αβ T cells. Our results demonstrate that the mechanisms involved in the proinflammatory effect of activated γδ T cells in Th17-mediated autoimmune responses include the binding of adenosine by activated γδ T cells and decreased CD73 expression on activated γδ T cells. Further studies on the role of adenosine in inflammation and immune responses should result in improved adenosine- and γδ T cell-based immunotherapies.

## Materials and Methods

All animal studies conformed to the Association for Research in Vision and Ophthalmology statement on the use of animals in Ophthalmic and Vision Research. Institutional approval by Institutional Animal Care and Use Committee (IACUC) of Doheny eye institute, University of Southern California was obtained and institutional guidelines regarding animal experimentation followed.

### Animals and reagents

Female C57BL/6 (B6) and TCR-δ^-/-^ mice on the B6 background, purchased from Jackson Laboratory (Bar Harbor, ME), were housed and maintained in the animal facilities of the University of Southern California. Recombinant murine IL-1, IL-7, and IL-23 were purchased from R & D (Minneapolis, MN). Fluorescein isothiocyanate (FITC)-, phycoerythrin (PE)-, or allophycocyanin (APC)-conjugated antibodies (Abs) against mouse CD73, CD44, αβ T cell receptor (TCR), or γδ TCR and their isotype control antibodies were purchased from Biolegend (San Diego, CA). (PE)-conjugated anti-mouse A2AR monoclonal antibody was purchased from Santa Cruz Biotechnology (Dallas, Texas). The non-selective AR agonist 50-N-ethylcarboxamidoadenosine (NECA), selective A2AR agonist 2-p-(2-carboxyethyl) phenethylamino-5′-N-ethylcarboxamidoadenosine (CGS21680), selective A2AR antagonist (SCH 58261), selective A2BR agonist (BAY 60-6538), and selective A2BR antagonist (MRS 1754), and the CD73 inhibitor α,β-methylene ADP (APCP) were purchased from Sigma-Aldrich (St. Louis, MO, USA).

### T cell preparations

αβ T cells were purified from B6 mice immunized with the human interphotoreceptor retinoid-binding protein (IRBP) peptide IRBP_1-20_, as described previously [22], [32], [33], while γδ T cells were purified from immunized and control (naïve) B6 mice. Nylon wool-enriched splenic T cells from naive or immunized mice were incubated sequentially for 10 min at 4°C with FITC-conjugated anti-mouse γδ TCR or αβ TCR Abs and 15 min at 4°C with anti-FITC Microbeads (Miltenyi Biotec GmbH, Bergisch Gladbach, Germany), then the cells were separated into bound and non-bound fractions on an autoMACS separator column (Miltenyi Biotec GmbH). The purity of the isolated cells, determined by flow cytometric analysis using PE-conjugated Abs against αβ or γδ T cells, was >95%.

Since the majority of αβ T cells isolated from immunized mice are non-activated [19], [22], activated αβ T cells were prepared by incubating the freshly isolated cells with anti-mouse CD3 Abs for 24 h at 37°C. In contrast, more than 60–80% of the γδ T cells isolated from immunized mice are already activated and express high levels of CD69 and IL-23R [19], [22]. Resting γδ T cells were prepared either from naïve mice or by incubating γδ T cells from immunized mice in cytokine-free medium for 5–7 days, at which time they show downregulation of CD69 expression [19]. Highly activated γδ T cells were prepared by incubating resting γδ T cells for 2 days with Abs against the γδ TCR (GL3) and CD28 (both 2 µg/ml, both from Bio-Legend, San Diego, CA), or cytokines combination (IL-1,IL-7 and IL-23).

### CFSE assay

Purified αβ T cells from IRBP_1-20_-immunized B6 mice were stained with CFSE (Sigma-Aldrich) as described previously [34]. Briefly, the cells were washed and suspended as 50×10^6^ cells/ml in serum-free RPMI 1640 medium (Corning Cellgro, VA), then were incubated at 37°C for 10 min with gentle shaking with a final concentration of 5 µM CFSE before being washed twice with RPMI 1640 medium containing 10% fetal calf serum (Atlantic Inc. Santa Fe, CA) (complete medium), suspended in complete medium, stimulated with immunizing peptide in the presence of irradiated syngeneic spleen cells as antigen-presenting cells (APCs), and analyzed by flow cytometry.

### Thymidine-incorporating proliferation assay

Purified αβ T cells (3×10^5^ cells/well) from IRBP_1-20_-immunized B6 mice in a total volume of 200 µl were cultured at 37°C for 48 h in 96-well microtiter plates in complete medium with or without 10 µg/ml of immunizing peptide in the presence of irradiated syngeneic spleen APCs (2×10^5^), and [^3^H] thymidine incorporation during the last 8 h was assessed using a microplate scintillation counter (Packard). The proliferative response was expressed as the mean cpm ± standard deviation (SD) of triplicate determinations.

### Adenosine binding assay

αβ or γδ T cells seeded in 96-well cell culture plates at a density of 1×10^5^/ml in 100 µl of complete medium were incubated at 37°C for 1 h with H^3^-adenosine at final concentrations of 0 to 12,000 nM in triplicate, then cell-bound and free H^3^-adenosine were separated by harvesting the cells on a cell harvester (Perkin Elmer) and the cell-associated radioactivity measured by liquid scintillation. Scatchard plot analysis was then performed and the dissociation constant and maximum binding capacity calculated.

### Measurement of adenosine receptor mRNA levels

A2AR and A2BR mRNA levels were determined by real-time PCR. αβ T cells, γδ T cells, dendritic cells (DCs), and B cells were purified from naive or IRBP_1-20_-immunized B6 mice by autoMACS separation. Total RNA was extracted from 2×10^5^ cells using an RNA isolation kit (Invitrogen, Carlsbad, CA) and treated with DNase I (GE Healthcare, Piscataway, NJ), then 0.1 µg was reverse transcribed into cDNA using a Moloney murine leukemia virus RT kit (Invitrogen) and tested in a Cyber green real-time PCR assay. Levels of each cDNA were measured in triplicate, using *Gapdh* cDNA as reference. Each cDNA sample was amplified for the gene of interest and β-actin (TaqMan assays; Mx3000P system; Stratagene, La Jolla, CA). The concentration of the mRNA for the gene of interest was determined using the comparative threshold cycle number and normalized to that of the internal *Gapdh* control, results were shown as 2^-△^Ct^^.

### Cytokine assays

Purified αβ T cells (3×10^4^ cells/well; 200 µl) from the draining lymph nodes and spleens of IRBP_1-20_-immunized B6 mice were cultured in complete medium at 37°C for 48 h in 96-well microtiter plates with irradiated syngeneic spleen APCs (1×10^5^) in the presence of 10 µg/ml of IRBP_1-20_, then a fraction of the culture supernatant was assayed for IL-17 and IFN-γ using ELISA kits (R & D).

### CD73 activity assay

αβ or γδ T cells were washed in Hank's balanced salt solution (HBSS) and suspended in HBSS at 1×10^6^ cells/ml, then 100 µl of the suspension was incubated for 1 h at 37°C with 1 mM adenosine monophosphate (AMP). The cells were then spun down and the supernatants diluted 10-fold with time-division multiplexing buffer (100 mM Tris, pH 7.4, 0.8 mM MgCl_2_, 1 mM mercaptoethanol) and analyzed for adenosine by HPLC. A reverse-phase HPLC column (Agilent Technologies, C18, particle size 5 µm, 250×4.6 mm) and a linear gradient of 0–50% methanol in water (1 ml/min) was used and the absorbance of the eluate monitored at 260 nm. The area under the adenosine peak was calculated using a computer program (Millennium Software), and the concentration of adenosine in the sample obtained by reference to a standard curve for adenosine.

### Statistical analysis

The results in the figures are representative of one experiment, which was repeated 3–5 times. The statistical significance of differences between groups in a single experimental was initially analyzed by ANOVA, and if statistical significance was detected the Student–Newman–Keuls post-hoc test was subsequently used. P values less than 0.05 was considered a statistically significant difference and marked with one *; when P<0.01, two ** were used.

## Results

### Ligation of the A2AR enhances γδ T cell responses

**Figure 1:**
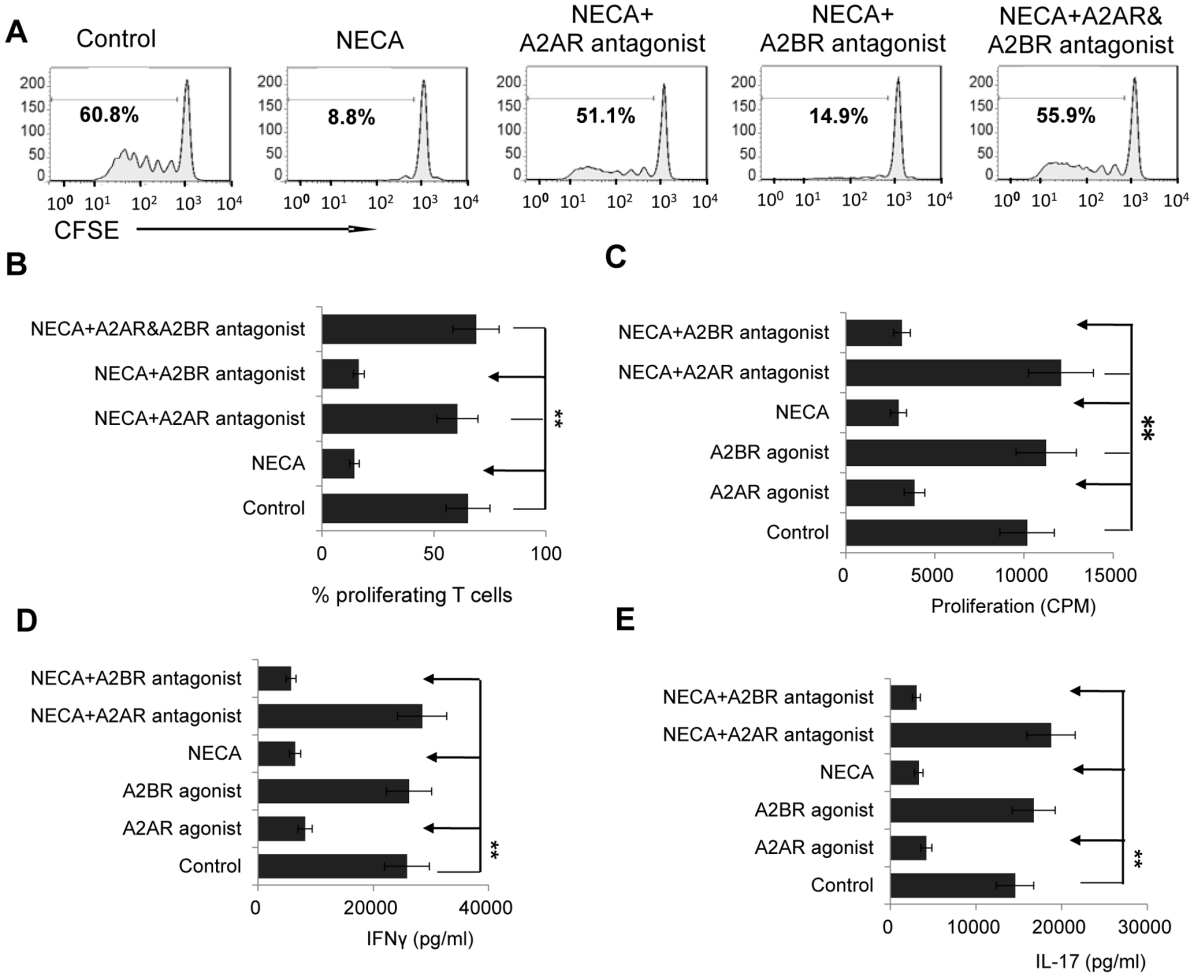
Ligation of the A2AR inhibits αβ T cell proliferative responses. A and B) CFSE-labeled cell proliferative assay. Responder αβ T cells isolated from immunized TCR-δ^-/-^ mice were labeled with CFSE, then incubated for 5 days with the immunizing peptide IRBP_1-20_ and syngeneic APCs in the presence or absence of the non-selective AR agonist NECA (100 nM) with or without the selective A2AR antagonist SCH 58261 (100 nM) and/or the selective A2BR antagonist MRS 1754 (100 nM). A shows a typical result and B the summarized data for 4 separate experiments. C). Thymidine incorporation assay. Splenic T cells isolated from immunized TCR-δ^-/-^ mice were stimulated for 48 h with the immunizing peptide IRBP_1-20_ and syngeneic APCs or APCs alone (control), with the selective A2AR agonist 2-p-(2-carboxyethyl) phenethylamino-5′-N-ethylcarboxamidoadenosine (CGS21680; 100 nM) or the selective A2BR agonist BAY 60-6538 (100 nM), or with NECA (100 nM) with or without the selective A2AR or A2BR antagonist (100 nM). The plates were then pulsed for 6 h with 0.5 µCi of [^3^H]thymidine/well and the cells assessed for isotope incorporation (Packard). D&E) Cytokine assay. IFN-γ (D) and IL-17 (E) levels in the supernatants from the cultures in (C) measured by ELISA. In all panels, the results shown are from a single experiment and are representative of those obtained in >5 experiments. **, p<0.01.

To determine the effect of adenosine on the autoimmune responses of IRBP-specific T cells, we immunized TCR-δ^-/-^ mice with the uveitogenic peptide IRBP_1-20_ and isolated total T cells from the immunized mice at day 13 post-immunization, when the highest number of in vivo primed T cells are present [19]–[21]. The T cells were then labeled with CFSE and subjected to antigenic stimulation with 10 µg/ml of immunizing peptide in the presence of APCs in the presence or absence of 100 nM 5′-N-ethylcarboxamidoadenosine (NECA), a non-selective AR agonist that binds to both A2ARs and A2BRs, with or without addition of the selective A2AR antagonist SCH 58261 and/or the selective A2BR antagonist MRS 1754 (both 100 nM) [35], [36]. The percentage of proliferating CFSE-labeled IRBP-specific T cells was significantly decreased by NECA (Fig. 1A and B) and this effect was inhibited by the selective A2AR antagonist, but not the A2BR antagonist (Fig. 1A and B), indicating that the suppressive effect was due to ligation of the A2AR. The proliferation results were confirmed using the ^3^H-thymidine incorporation assay (Fig. 1C), which also showed that the effect of NECA was mimicked by the selective A2AR agonist 2-p-(2-carboxyethyl) phenethylamino-5′-N-ethylcarboxamidoadenosine (CGS21680), but not by the selective A2BR agonist BAY 60-6538. The results of cytokine assays on the supernatants from the same cultures agreed with the proliferation results by showing that production of IFN-γ (Fig. 1D) and IL-17 (Fig. 1E) was significantly decreased in the presence of NECA or the selective A2AR agonist, but not the selective A2BR agonist, and that the effect of NECA was inhibited by the selective A2AR antagonist, CH 58261, but not by the selective A2BR antagonist MRS 1754.

**Figure 2:**
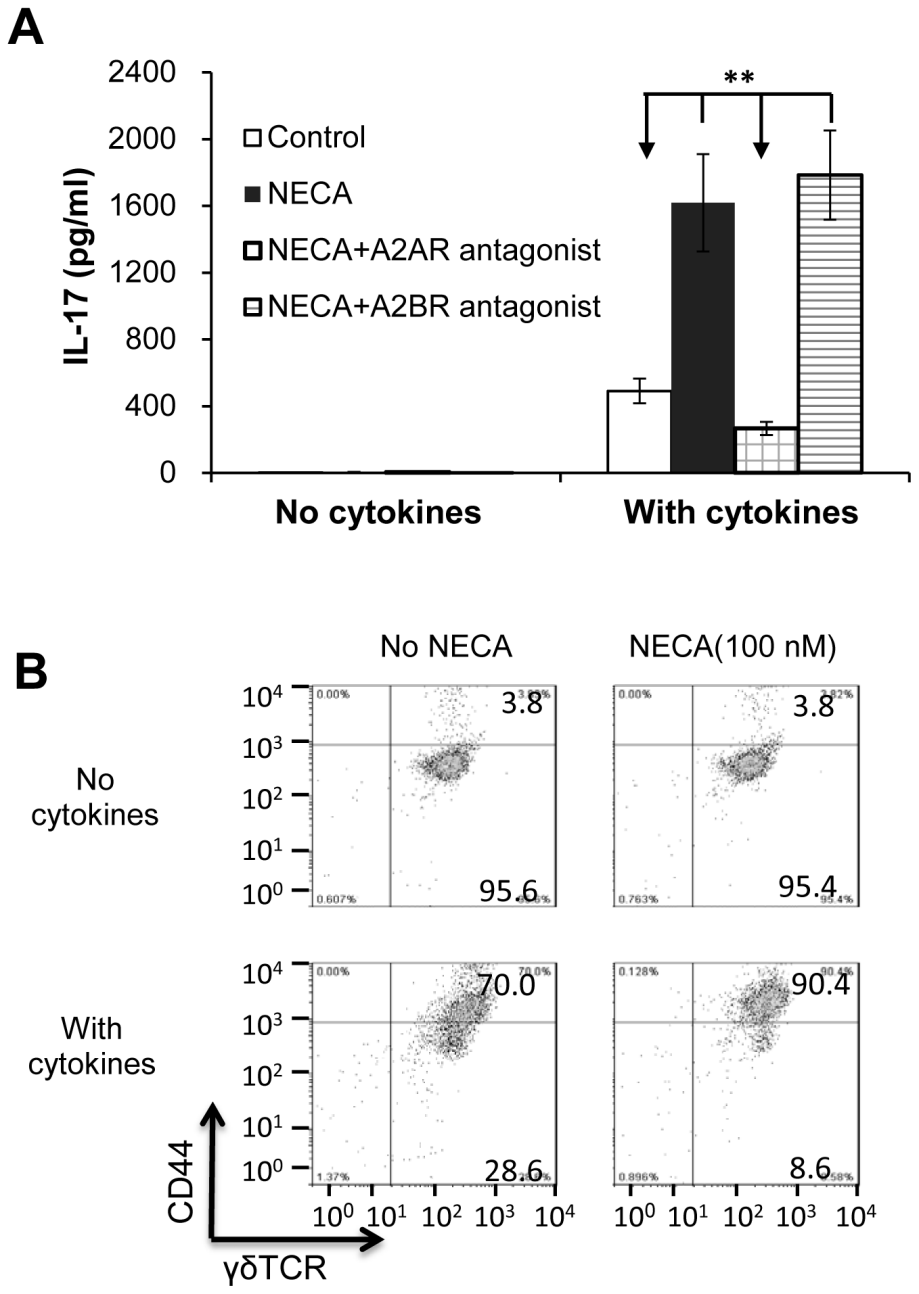
A2AR signaling enhances γδ T cell activation by a cytokine mixture. IL-17 production by γδ T cells. γδ T cells purified from immunized B6 mice (see M&M) were rested by incubating in cytokine-free medium for 5–7 days before testing. In 24-well plate, 2×10^5^ γδ T cells were incubated 48h alone (Control) or with NECA (100 nM) with or without the A2AR or A2BR antagonist (100 nM) in the absence (left panel) or presence (right panel) of the cytokine mixture (IL-1, IL-7, and IL-23). **, p<0.01. Surface staining for the T cell activation marker CD44 showing that rested γδ T cells express low amounts of CD44, which is significantly increased by incubation 48h with the cytokine mixture and further increased when γδ T cells are exposed to cytokines in the presence of NECA. The results shown are from a single experiment and are representative of those obtained in >5 experiments.

We then assessed the effect of NECA on γδ T cell activation. γδ T cells were prepared from immunized B6 mice, rested for a few days by culture in cytokine-free medium [19]–[21], then tested for activation by cytokines in the presence or absence of NECA with or without the A2AR or A2BR antagonist. Since we have previously shown that purified γδ T cells can be activated by a number of proinflammatory cytokines and that a mixture of IL-1, IL-7, and IL-23 has a strong stimulatory effect [22], we used this combination in this study. As shown in Fig. 2A, the AR agonist NECA did not itself stimulate IL-17 production by γδ T cells, but significantly enhanced IL-17 production induced by the cytokine mixture, and this effect was blocked by the A2AR antagonist, but not the A2BR antagonist. Furthermore, as shown in Fig, 2B, resting γδ T cells expressed low levels of the T cell activation marker CD44 in the presence or absence of NECA in the absence of added cytokines (upper panels), but levels were significantly increased by cytokine treatment and further increased in the presence of NECA (lower panels). A similar synergistic effect was seen when γδ T cells were exposed to a combination of a TLR ligand and NECA (not shown).

### Increased AR2R expression in activated γδ T cells

**Figure 3:**
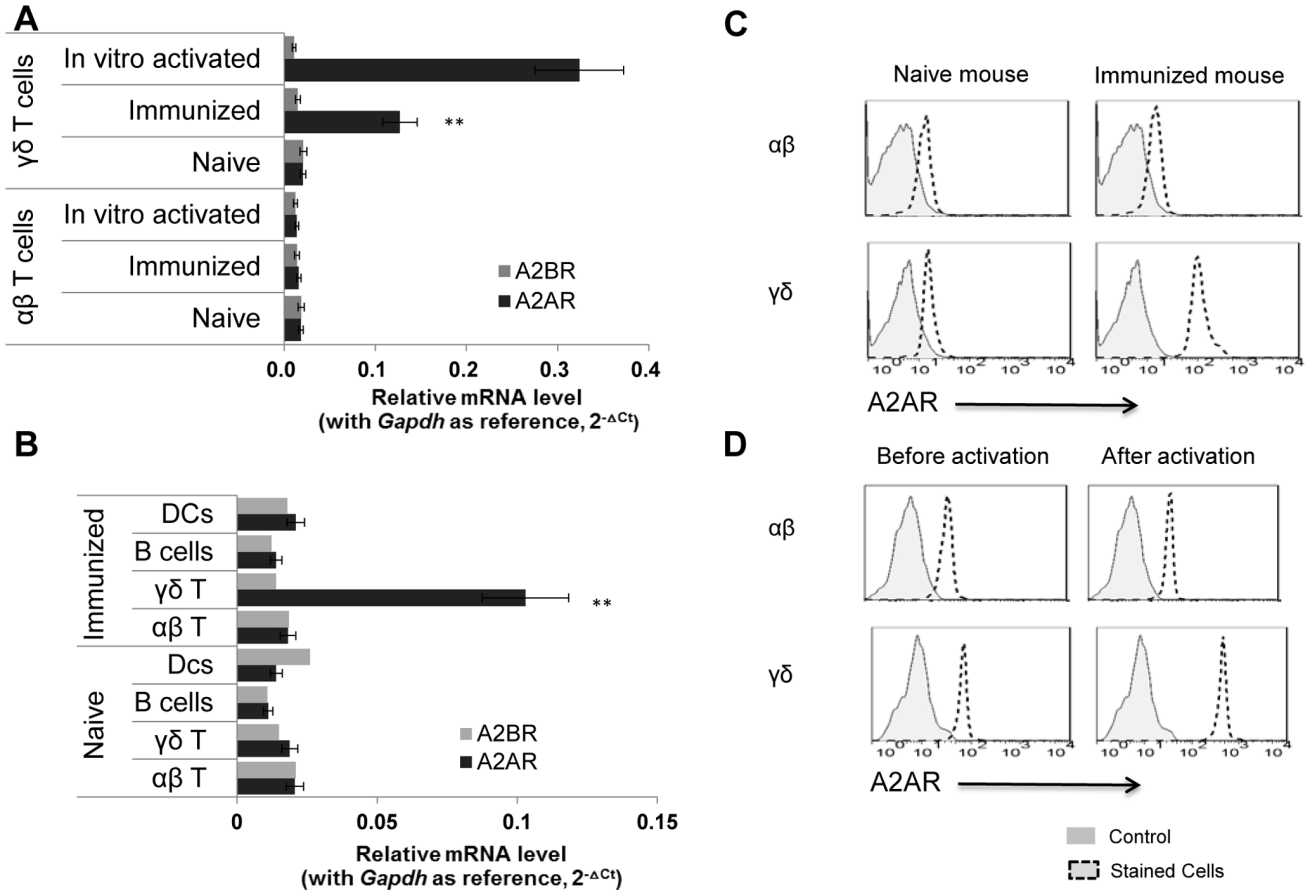
Activated γδ T cells expressed high levels of A2AR. Real-time RT-PCR analysis of A2AR and A2B transcripts among total RNA isolated from αβ and γδ T cells isolated from naïve and IRBP_1-20_-immunized B6 mice before and after in vitro activation. qPCR was performed with Gapdh as the internal reference. Results were represented as 2^-△Ct^. **, p<0.01. A2AR and A2BR mRNA levels in B cells DCs, αβ and γδ T cells. B cells and DCs were purified from splenocytes and drainage lymphocytes of naïve and immunized B6 mice by auto-MACs purification, using (PE)-conjugated anti- B220, anti- CD11c antibodies and anti-PE antibodies conjugated magnetic beads. A2AR and A2BR mRNA levels in B cells, DCs, αβ and γδ T cells were measured by real-time PCR. **, p<0.01. and D) Flow cytometry comparison of A2AR expression between αβ and γδ T cells. Figure 3C compares the αβ and γδ T cells freshly prepared from naïve or IRBP_1-20_-immunized B6 mice. Figure 3D compares the cultured αβ and γδ T cells before or after an in vitro activation by anti-CD3 antibody. All the cells were stained with a PE-conjugated anti-mouse A2AR antibody followed by FACS analysis. The controls were stained with an irrelevant PE-labeled antibody.

To understand the different effects of adenosine on IRBP-specific αβ and γδ T cells, we measured A2AR and A2BR mRNA levels in αβ and γδ T cells from naive and immunized mice using real-time PCR. Fig. 3A shows that both αβ T cells and γδ T cells from naïve mice contained low levels of A2AR and A2BR mRNAs, while γδ T cells, but not αβ T cells, from immunized mice contained significantly higher levels of A2AR mRNA, but not A2BR mRNA. After in vitro activation with anti-CD3 Ab or the cytokine mixture, A2AR expression was further increased on γδ T cells, but not αβ T cells, from immunized mice. Figure 3B, which compares A2AR and A2BR mRNA levels in T cells and other types of immune cells (B cells and DCs) from naïve (lower panel) or immunized (upper panel) mice, shows that γδ T cells from immunized mice expressed the highest A2AR mRNA levels and that the low levels in the other cell types were not significantly increased by immunization. We have also compared surface expressed A2AR between αβ and γδ T cells freshly isolated from naïve or immunized mice (Figure 3C) and among αβ and γδ T cells before or after in vitro activation (Figure 3D), using FACS analysis. As shown, γδ T cells from immunized mice expressed significantly increased A2AR as compared to the γδ T cells isolated from naïve mouse and to the αβ T cells from immunized mice (Figure 3C). Upon an in vitro exposure (48 h) to annti-CD3 antibody (1 µg/ml), αβ T cells expressed slightly increased A2AR, whereas γδ T cells express greatly increased numbers of A2AR (Figure 3D) compared to the same cells that were not activated.

### Increased adenosine-binding ability correlates with increased A2AR expression in *in vivo* activated γδ T cells

**Figure 4:**
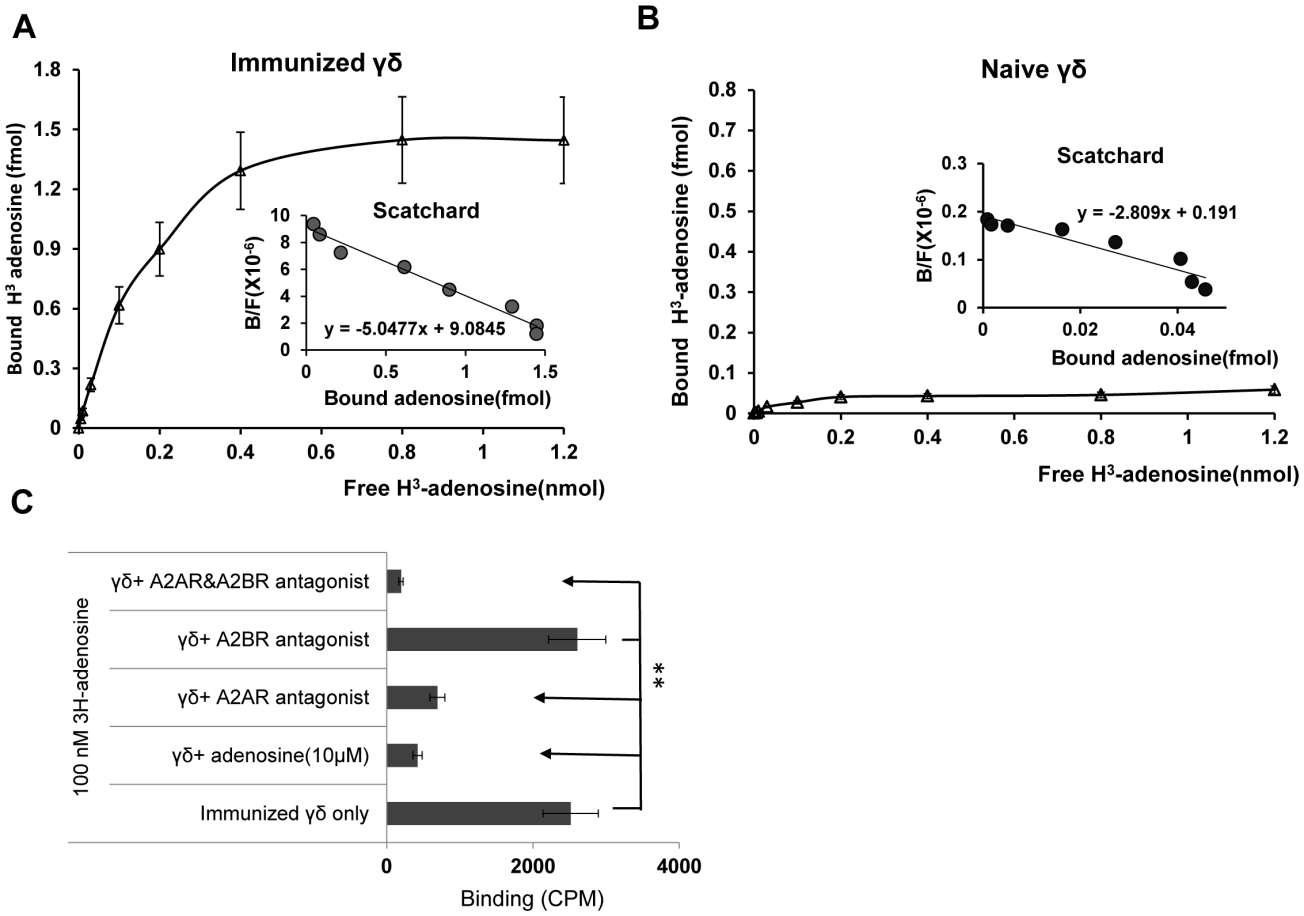
Activated γδ T cells show increased ability to bind adenosine. A–B) 1×10^5^ γδ T cells from IRBP_1-20_-immunized (A) or naïve (B) B6 mice were incubated with 0 to 12,000 nM H^3^-labeled adenosine for 1 h, then the cells were harvested and cell-bound H^3^-adenosine counted by liquid scintillation. C). A2AR is the major AR on γδ T cells from immunized B6 mice. In 96-well plate γδ T cells (1×10^5^/well) from IRBP_1-20_-immunized mice were incubated with 100 nM radiolabeled adenosine alone or after 1 h preincubation with 10 µM NECA or 100 nM A2AR antagonist (SCH 58261) and/or A2BR antagonist (MRS 1754). ** p<0.01.

To determine whether increased A2AR mRNA levels correlated with increased adenosine-binding activity, we compared the adenosine-binding activity of γδ T cells from naïve and immunized mice using a radioactive ligand binding assay (described in the Materials and Methods section). As shown in Fig. 4A and B, γδ T cells from immunized mice bound much higher levels of adenosine than γδ T cells from naive mice. Scatchard plot analysis of the reversible ligand/receptor binding interaction (insets in Fig. 4A and B) showed a straight line, with a slope -K that represents the affinity constant for ligand binding, while the maximum binding capacity for the ligand (Bmax) was determined from the intercept on the × axis. The K_D_ values for naive and immunized γδ T cells for adenosine were 0.36 and 0.20 nM, respectively, while the Bmax values were 0.07 and 1.8 fmol. Moreover, the binding of H^3^-labeled adenosine by γδ T cells plateaued at high adenosine concentrations (Fig. 4A and B), and binding of 100 nm H^3^-labeled adenosine was blocked by preincubation of the cells with 10 µM NECA or 100 nM A2AR antagonist, but not A2BR antagonist (Fig. 4C), indicating that binding was saturable and mediated by A2ARs.

### The inhibitory effect of an AR agonist on αβ T cell activation is reduced in the presence of A2AR^+^ γδ T cells

**Figure 5:**
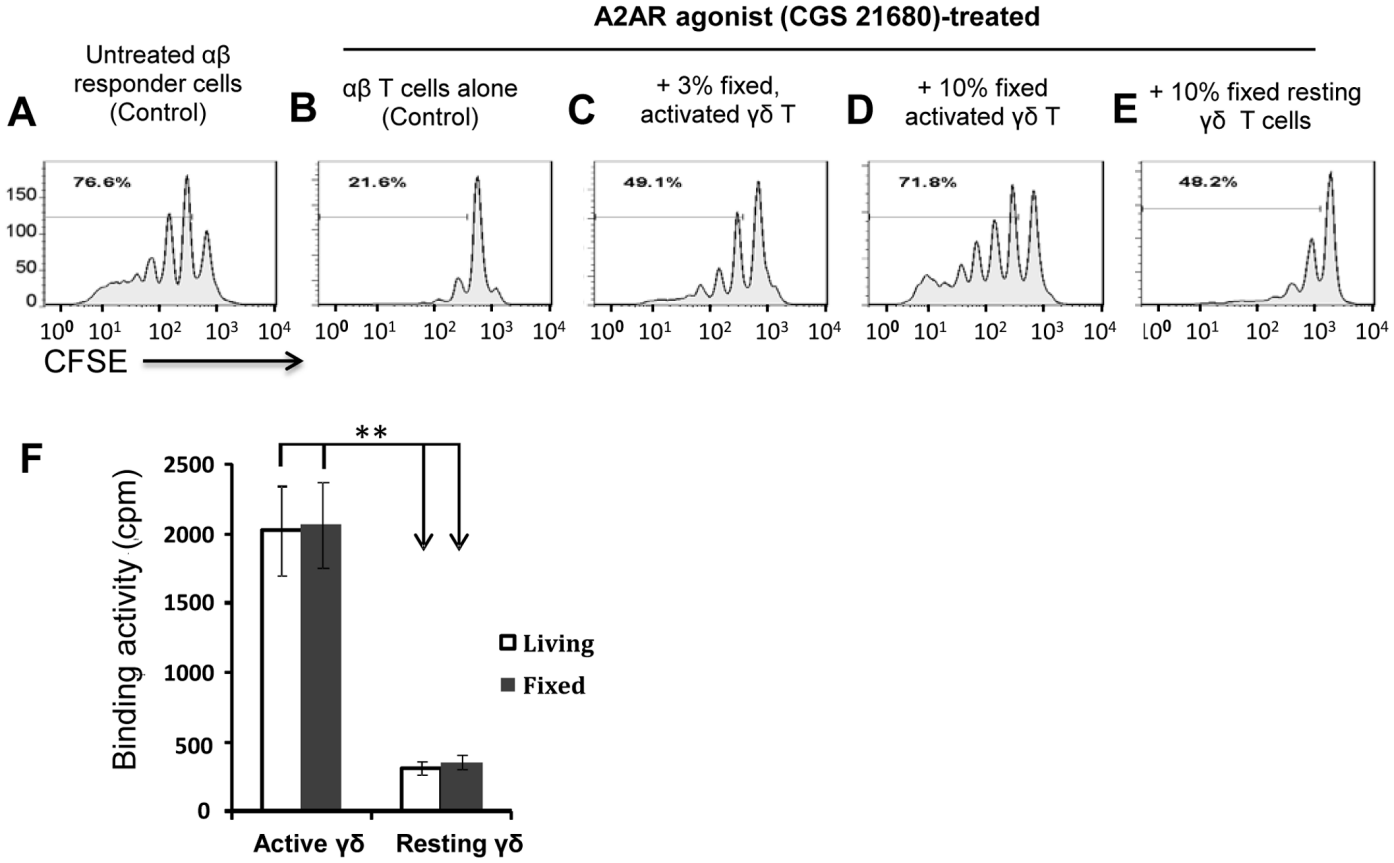
The suppressive effect of an A2AR agonist on the proliferation of CFSE-labeled αβ T cells is decreased in the presence of A2AR^+^ γδ T cells. A–E) in 24-well plate, αβ responder T cells (1×10^6^/well] isolated from IRBP_1-20_-immunized TCR-δ^-/-^ mice were labeled with CFSE before incubation for 5 days with immunizing peptide in the presence of APCs alone (A) or in the presence of the A2AR agonist CGS 21680 (100 nM) (B–E) in the absence of γδ T cells (B) or in the presence of 3% (C) or 10% (D) fixed activated γδ T cells or 10% fixed resting γδ T cells from immunized B6 mice, then proliferating cells were measured by FACS analysis. F) Formalin-fixed activated γδ T cells are as effective as activated live cells in binding adenosine. **, p<0.01.

To examine whether the increased adenosine binding activity of activated γδ T cells correlated with their immunoregulatory function, we assessed the effect of an A2AR agonist, CGS 21680 [3], [37], on the proliferation of CFSE-labeled αβ T cells in the absence or presence of in vitro activated γδ T cells, generated as described in the Methods. CFSE-labeled αβ responder T cells isolated from immunized TCR-δ^-/-^ mice were incubated for 5 days with the immunizing peptide and APCs in the presence or absence of a small percentage of fixed resting or activated γδ T cells from immunized B6 mice, then αβ T cell proliferation was assessed by FACS analysis. As shown in Fig. 5A-D, addition of the A2AR agonist CGS 21680 significantly inhibited the proliferation of CFSE-labeled αβ responder T cells (compare B with A) and this effect was significantly inhibited by addition of 3% of formalin-fixed γδ T cells from immunized B6 mice (C) or completely blocked by addition of 10% of fixed activated γδ T cells (D). In addition, activated γδ T cells from immunized B6 mice were far more effective (D) than resting γδ T cells (E) in neutralizing the suppressive effect of CGS 21680. Fixation of activated γδ T cells had no effect on the binding of radiolabeled adenosine (Fig. 5F).

### Activated γδ T cells express decreased amounts of CD73

**Figure 6:**
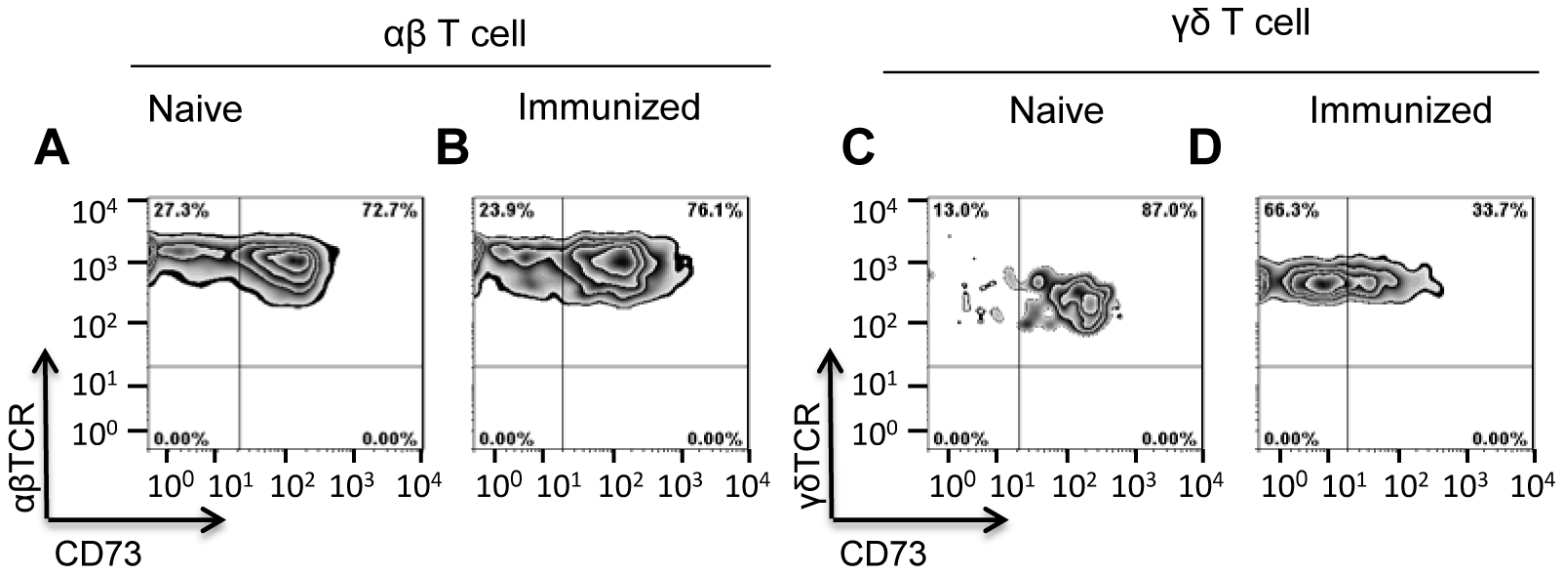
Activated γδ T cells, but not αβ T cells, express decreased levels of CD73. αβ (A &B) and γδ T cells (C &D), prepared from either naïve or immunized B6 mice, as described in the Materials and Methods, were double-stained with PE-labeled anti-mouse γδ TCR or αβ TCR antibodies and APC-labeled anti-mouse CD73 antibodies and analyzed by cytometry.

**Figure 7:**
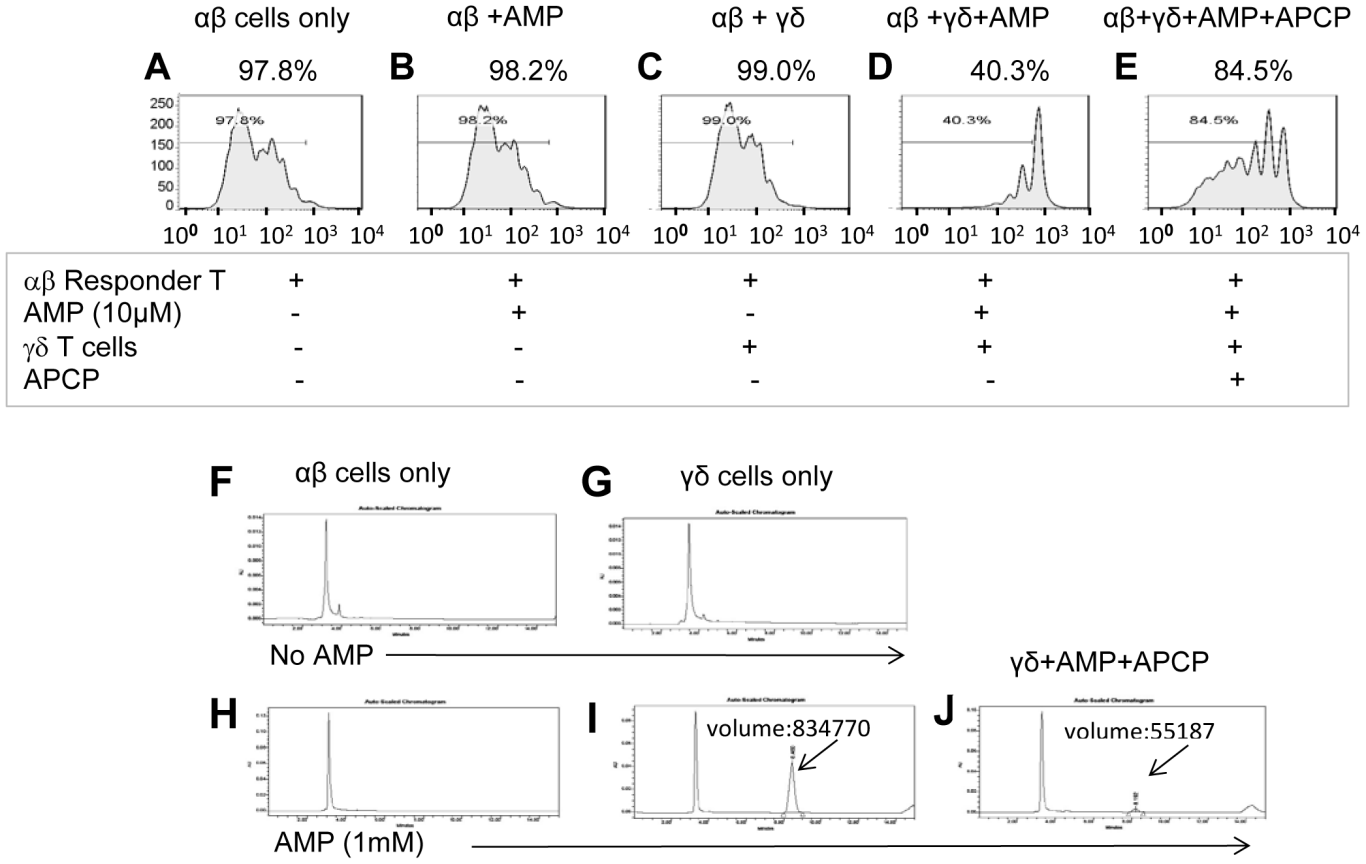
AMP inhibits the αβ T cell proliferative response only in the presence of γδ T cells and the response is inhibited by the CD73 inhibitor APCP. (A–E) Proliferative responses of CFSE-labeled αβ T cells isolated from immunized TCR-δ^-/-^ B6 mice (1×10^5^/well) assessed after in vitro incubation for 1 h with immunizing peptide and APCs alone (A) or in the presence of 10 µM AMP (B), 5% resting γδ T cells (C), AMP and γδ T cells (D), or AMP and γδ T cells and 3 µM APCP (E) in 96 well plate. The results shown are representative of those obtained in three separate experiments. (F–J) HPLC analysis showing the amount of adenosine in culture supernatants after 1 h culture of αβ T cells (3×10^5^/well) (F), γδ T cells (1×10^5^/well) (G), αβ T cells+1 mM AMP (H), γδ T cells+1 mM AMP (I), or γδ T cells+1 mM AMP+3 µM APCP (J) in 96 well plate. The adenosine peak is indicated by the arrow. Results shown are from a single experiment and are representative of three experiments.

The ecto-enzyme CD73 (ecto-5'-nucleotidase) converts immunostimulatory AMP into immunosuppressive adenosine [10], [31]. As shown in Fig. 6, 72.7% of resting αβ T cells (A) and 87% γδ T cells (C) from naive B6 mice expressed CD73. In contrast, only a low percentage (34%) of the γδ T cells from immunized B6 mice (D) (or in vitro activated γδ T cells, data not shown) expressed low levels of CD73, whereas activation of αβ T cells from immunized mice (B) did not significantly alter CD73 expression, as compared to the same cells from naïve mice (A). As shown in Fig 7A–E, 10 nM AMP had no inhibitory effect on the proliferative response of CSFE-labeled αβ responder T cells unless a small percentage (5%) of resting γδ T cells was also present, and this inhibitory effect of γδ T cells was markedly inhibited by the CD73 inhibitor APCP.

To determine whether the suppressive effect on αβ T cell proliferation correlated with the amount of adenosine generated, we compared the generation of adenosine from AMP in the supernatants of such cultures using HPLC. No adenosine was detectable in the supernatants of αβ or γδ T cells cultured in the absence of exogenously added AMP (Fig. 7F and G), while, after incubation with 1 mM AMP for 1 h, an adenosine peak (indicated by the arrow) was seen in the γδ T cell cultures (I), but not the αβ T cell cultures (H), and this peak was much smaller when the CD73 inhibitor APCP was added to the cultures (J). Since αβ and γδ T cells express comparable levels of CD73 (Fig. 6A and B), we conclude that CD73 on αβ T cells is less able to convert AMP into immunosuppressive adenosine.

## Discussion

Adenosine is an endogenous purine nucleoside that modulates a wide range of physiological functions [4], [38], including immune system function [39]. It is released in the vicinity of immune cells in tissues subjected to various forms of injury, including ischemia and inflammation. Under physiological conditions, only low concentrations of adenosine are present in the extracellular space, but levels increase dramatically under stressful conditions [2]. Adenosine has been shown to play an important role in tumor growth [10], [31], [40], [41] and inflammation [3], [6], [12], [13], [39], [42], [43]. Although adenosine exerts its functions by binding to four different ARs, designated A1R, A2AR, A2BR, and A3R [3], [5], [23], [24], it has long been recognized that it suppresses T cell activity primarily by acting on A2ARs [9], [25]–[29]. In efforts to determine which cells are most affected by adenosine, many types of immune cells have been studied, including T cells [10], [11], [42], [44], [45], macrophages/DCs [9], [36], [46], NK cells [47], neutrophils [24], [48], platelets [49], and regulatory T cells (Treg) [6], [9], [50]; however, the role of adenosine and ARs in γδ T cell function has not been extensively studied. Given that γδ T cells are one of the major inflammatory cells invading inflamed organs during inflammation [51]–[53] and have a regulatory effect on various immune responses [51], [54]–[57], including Th17 autoreactive T cell responses [19], [20], and since adenosine affects regulatory T cell functions [6], [50], [58], [59], we examined whether adenosine had an effect on γδ T cell-mediated immunoregulation. Our results showed that adenosine can be added to the list of molecules that modulate γδ T cell function. Moreover, our results showed that adenosine was not a strong γδ T cell stimulator on its own, but significantly enhanced the γδ T cell-stimulating effect of cytokines (Fig. 2) and TLR ligands (data not shown). Adenosine modulators might therefore be useful tools for restraining γδ T cell activation and thus the activation of Th17 autoreactive responses.

We have previously shown that γδ T cells in immunized mice are partially activated and that their function differs greatly from that of their non-activated counterparts [19], [22]. Under different pathogenic conditions, γδ T cells can be activated by various factors, such as tetanus toxoid [60], staphylococcal enterotoxin A [61], heat shock protein 65 [62], isopentenyl pyrophosphate [63], and IL-1β plus IL-23 [64], Since adenosine is also frequently generated under various pathogenic conditions, it is likely that adenosine is more effective in increasing γδ T cell activation in an inflammatory environment, as it can act synergistically with other stimulatory molecules (Fig. 2A). In addition, γδ T cells from immunized mice are partially activated and possess significant proinflammatory activity, which can be further enhanced by an in vitro activation, leading to a further augmentation of their proinflammatory effect [19], [21].

In this study, we showed that the inhibitory effect of the A2AR agonist CGS 21680 on the proliferation of autoreactive αβ T cells was markedly inhibited by the presence of a small percentage (3–10%) of activated γδ T cells (Fig. 5) and that this neutralizing effect was positively correlated with A2AR mRNA expression in γδ T cells, as it was not seen using resting γδ T cells (Fig. 5), which express less A2AR mRNA than activated γδ T cells (Fig. 3). A binding assay showed that activated γδ T cells bound far more adenosine than other immune cells (data not shown). The inhibitory effect of the AR agonist on the proliferative response of αβ T cells was markedly inhibited by a specific A2AR antagonist, but not an A2BR antagonist (Fig. 1A–C). We also showed that activated γδ T cells (A2AR^high^) bound much more radiolabeled adenosine than non-activated γδ T cells (Fig. 4). Our results suggest that increased A2AR expression allows γδ T cells to bind adenosine in the inflamed tissue, thus preventing its suppressive effect on αβ T cells, leading to an enhanced immune response. However, strong binding of adenosine by activated γδ T cells works more than a ”sink” effect; for example, binding of adenosine by γδ T cells enhanced γδ activation rendering them more competitive in adenosine binding. Since activated γδ T cells have a strong ability to enhance the Th17 response, the binding of adenosine by these γδT cells may have also weakened the suppressive effect of adenosine on αβ T cells.

Our results also showed that activated γδ T cells expressed decreased levels of CD73, an ecto-enzyme responsible for the conversion of immunostimulatory AMP into immunosuppressive adenosine [10], [31]. Thus, activation of γδ T cells not only alters adenosine-mediated immunoregulation, but also adenosine metabolism. To test the in vivo effect of AR agonist on EAU, we have finished an in vivo study (paper submitted to Journal of Immunology), which demonstrated that injection of mice with NECA at an early stage after immunization but prior to ocular inflammation had an inhibitory effect on both Th1 and Th17 responses leading to disease suppression, which supported the recent observation that AR agonist had an inhibitory effect on EAU [65]. However, we also showed that the agonist effect on the Th17 response is different compared to the Th1 response. When the same amount of agonist is injected at a late (inflammatory) stage, it enhanced rather than inhibited the Th17 response. Thus, our in vivo data agreed with the in vitro study presented here that proinflammatory cytokine can convert inhibitory effect of AR agonist on Th17 response.

In a recent study [22], we demonstrated that activated γδ T cells express high levels of the IL-23R and that binding of IL-23 by activated γδ T cells removes IL-23 required for the activation of Th17 autoreactive T cell responses. In the present study, we showed that A2AR^+^ γδ T cells enhanced the adaptive response by binding adenosine, indicating that γδ T cells are both able to suppress and enhance autoimmune responses, depending on conditions in the microenvironment. Given our previous finding that activated γδ T cells can promote the activation of Th17 autoreactive T cell responses in experimental autoimmune uveitis [19], [22], [33], we predict that increased generation of adenosine does not necessarily result in suppression of immune responses and that high adenosine levels may lead to enhanced γδ T cell activation, which, in turn, will augment Th17 responses. Since the γδ T cell-stimulating effect of the AR agonist NECA was only seen in the presence of additional γδ T cell activation stimulators, such as proinflammatory cytokines and TLR ligands (Fig. 2A), we suggest that adenosine exerts a suppressive function in microenvironments lacking proinflammatory factors, whereas, in microenvironments rich in proinflammatory cytokines and TLR ligands, its suppressive effect is converted into a proinflammatory effect, though such a prediction remains to be further tested. The continuation of the study should reveal the mechanisms by which adenosine inhibits or promotes an immune response during different disease phases and may lead to a more effective therapies.
